# Comparative Evaluation of the Fracture Resistance of Fractured Teeth Reattached Using Different Preparation and Restoration Techniques: An In Vitro Study

**DOI:** 10.7759/cureus.114843

**Published:** 2026-08-20

**Authors:** Sagar Saxena, Charu Dayal, Rishi Manan, Bharat Chauhan, Digvijay Singh, Parul Bansal

**Affiliations:** 1 Department of Conservative Dentistry and Endodontics, Institute of Dental Studies and Technologies, Modinagar, IND

**Keywords:** composite resins, dental restoration, fracture resistance, tooth fractures, tooth reattachment

## Abstract

Introduction: The restoration of fractured anterior teeth remains a clinical challenge because an ideal technique should preserve tooth structure while providing adequate mechanical strength and esthetic outcomes. Although fragment reattachment is a conservative approach, various preparation designs have been proposed to improve fracture resistance, and evidence comparing their performance remains limited. Our aim was to evaluate and compare the fracture resistance of Ellis Class II fractured maxillary central incisors restored using bevel preparation, single groove preparation, double-groove preparation, and direct composite build-up techniques.

Methodology: This in vitro experimental study included 40 extracted permanent maxillary central incisors, which were randomly allocated into four groups (*n* = 10 each). Standardized Ellis Class II fractures were created, followed by restoration using bevel reattachment, single groove reattachment, double-groove reattachment, or direct composite build-up. All specimens were restored using a universal adhesive system and microhybrid composite resin. After finishing and polishing, fracture resistance was evaluated using a universal testing machine at a crosshead speed of 1 mm/minute. Data were analyzed using one-way analysis of variance, followed by Tukey's post hoc test, with statistical significance set at *P* < 0.05.

Results: Fracture resistance differed significantly among the four groups (*P* < 0.001). The direct composite build-up group demonstrated the highest mean fracture resistance (239.23 ± 56.61 N), followed by the double-groove (198.60 ± 28.40 N), single-groove (169.94 ± 43.52 N), and bevel (134.53 ± 28.85 N) groups. Pairwise analysis showed that the fracture resistance of the direct composite build-up was significantly higher than that of the bevel and single groove techniques, whereas the double-groove technique exhibited significantly greater fracture resistance than the bevel preparation.

Conclusions: Direct composite build-up exhibited the highest fracture resistance among the evaluated restorative techniques. Among the fragment reattachment methods, double-groove preparation provided superior reinforcement and may represent the preferred conservative technique when a fractured fragment is available.

## Introduction

Traumatic dental injuries are among the most common emergencies in restorative dentistry, with coronal fractures of the anterior teeth accounting for the majority of cases [1]. Maxillary central incisors are particularly susceptible to fractures because of their prominent position within the dental arch and exposure to direct impact during falls, sports-related injuries, road traffic accidents, and interpersonal violence [2]. These injuries are prevalent among children, adolescents, and young adults and often result in compromised aesthetics, function, phonetics, and psychological well-being. In addition to the immediate structural damage, anterior tooth fractures may adversely affect self-confidence and social interactions, highlighting the importance of prompt and conservative management [3].

Advances in adhesive dentistry have transformed the management of fractured anterior teeth in recent years. Earlier restorative approaches, including full-coverage crowns and extensive composite restorations, frequently required the removal of sound tooth structure [4,5]. Contemporary adhesive systems and resin composites have enabled minimally invasive restorative procedures that preserve healthy dental tissues while providing satisfactory esthetic and functional outcomes. When the fractured fragment is unavailable, direct composite build-up remains the treatment of choice; however, challenges such as polymerization shrinkage, marginal discoloration, wear, and long-term bond degradation may compromise clinical longevity [6].

When a fractured tooth fragment is available and intact, fragment reattachment is considered one of the most conservative treatment options. This technique preserves the natural enamel, dentin, surface texture, translucency, and occlusal anatomy while maintaining the maximum tooth structure [7,8]. Various preparation designs, including bevels, internal grooves, and multiple groove configurations, have been proposed to improve the bonding surface area, stress distribution, and fracture resistance of posts [7]. Nevertheless, there is no consensus on the preparation technique that provides the greatest reinforcement after fragment reattachment, necessitating further laboratory evaluation.

This in vitro study aimed to evaluate and compare the fracture resistance of Ellis Class II fractured maxillary central incisors restored using different preparation and restoration techniques. The objectives of this study were to evaluate the fracture resistance of teeth restored using bevel, single-groove, double-groove, and direct composite build-up techniques and to compare the fracture resistance among these four restorative approaches to identify the technique that provides the greatest reinforcement of fractured teeth. The null hypothesis was that there would be no significant difference in fracture resistance among maxillary central incisors restored using bevel preparation, single-groove preparation, double-groove preparation, and direct composite build-up techniques.

## Materials and methods

Study design, setting, duration, and ethical considerations

This in vitro experimental study was conducted in the Department of Conservative Dentistry and Endodontics, Institute of Dental Studies and Technologies, Modinagar, Uttar Pradesh, India, over a period of six months (May 2023 to October 2023). As the investigation involved only extracted human teeth obtained following routine therapeutic extractions, no patient interventions were performed. Written informed consent was obtained from the patients permitting the use of extracted teeth for research purposes. The study protocol was approved by the Institutional Ethics Committee of the college (Approval No.: IDST/IEC/2022-25/05).

Sample size calculation

The required sample size was estimated using G*Power software (version 3.1.9.7; Heinrich Heine University, Düsseldorf, Germany) based on the fracture resistance values reported by Abdulkhayum et al. [9]. A one-way analysis of variance (ANOVA) model was considered with an effect size (*f*) of 0.55, significance level (α) of 0.05, and statistical power of 80%. The analysis indicated that a minimum of 40 specimens is required. Forty extracted maxillary central incisors were included and equally allocated into four study groups, each consisting of 10 specimens. Maxillary central incisors were selected because they are highly relevant to anterior crown-fracture reattachment and provide relatively uniform morphology for standardized preparation and fracture testing.

Eligibility criteria

Freshly extracted permanent maxillary central incisors for periodontal reasons with intact crowns and fully developed roots were included in this study. Teeth exhibiting dental caries, restorations, developmental anomalies, cracks, fractures, root resorption, severe attrition, erosion, abrasion, or previous endodontic treatment were excluded from the study. Following collection, all specimens were thoroughly cleaned using hand scalers to remove residual periodontal tissue and calculus and were stored in 0.9% normal saline at room temperature until use to prevent sample dehydration.

Specimen preparation and restorative procedures

Forty extracted permanent maxillary central incisors fulfilling the eligibility criteria were cleaned of adherent soft tissue and calculus using hand scalers and stored in 0.9% normal saline at room temperature until experimentation to prevent dehydration. The roots of all specimens were embedded vertically in self-curing acrylic resin blocks, leaving the clinical crowns exposed and aligning the long axis of each tooth perpendicular to the acrylic base. To standardize the fracture creation, the labial surface of each tooth was visually divided into longitudinal and transverse thirds, and an oblique Ellis Class II crown fracture extending from the proximal surface to the incisal edge was created using a diamond disc mounted on a straight handpiece (Waldent Innovations Pvt. Ltd., New Delhi, India) under continuous water irrigation [10]. Following sectioning, the fractured fragments and remaining tooth structures were immediately stored in normal saline until restoration.

The specimens were randomly allocated to four equal groups (*n* = 10 each) using a computer-generated randomization sequence. In the first group (bevel group), a circumferential 45° bevel approximately 1 mm wide was prepared on both the fractured tooth and the detached fragment using a flame-shaped diamond finishing bur before fragment reattachment. In the second group (single-groove group), a single internal groove measuring approximately 1 mm in depth, width, and length was prepared centrally along the fracture interface to improve mechanical retention. In the third group (double-groove group), two standardized grooves of identical dimensions were prepared symmetrically along the fracture line of the teeth. In the fourth group (composite build-up group), the fractured fragment was discarded, and the missing tooth structure was restored directly with composite resin after preparing a 45° bevel on the fractured enamel margins.

**Figure 1:**
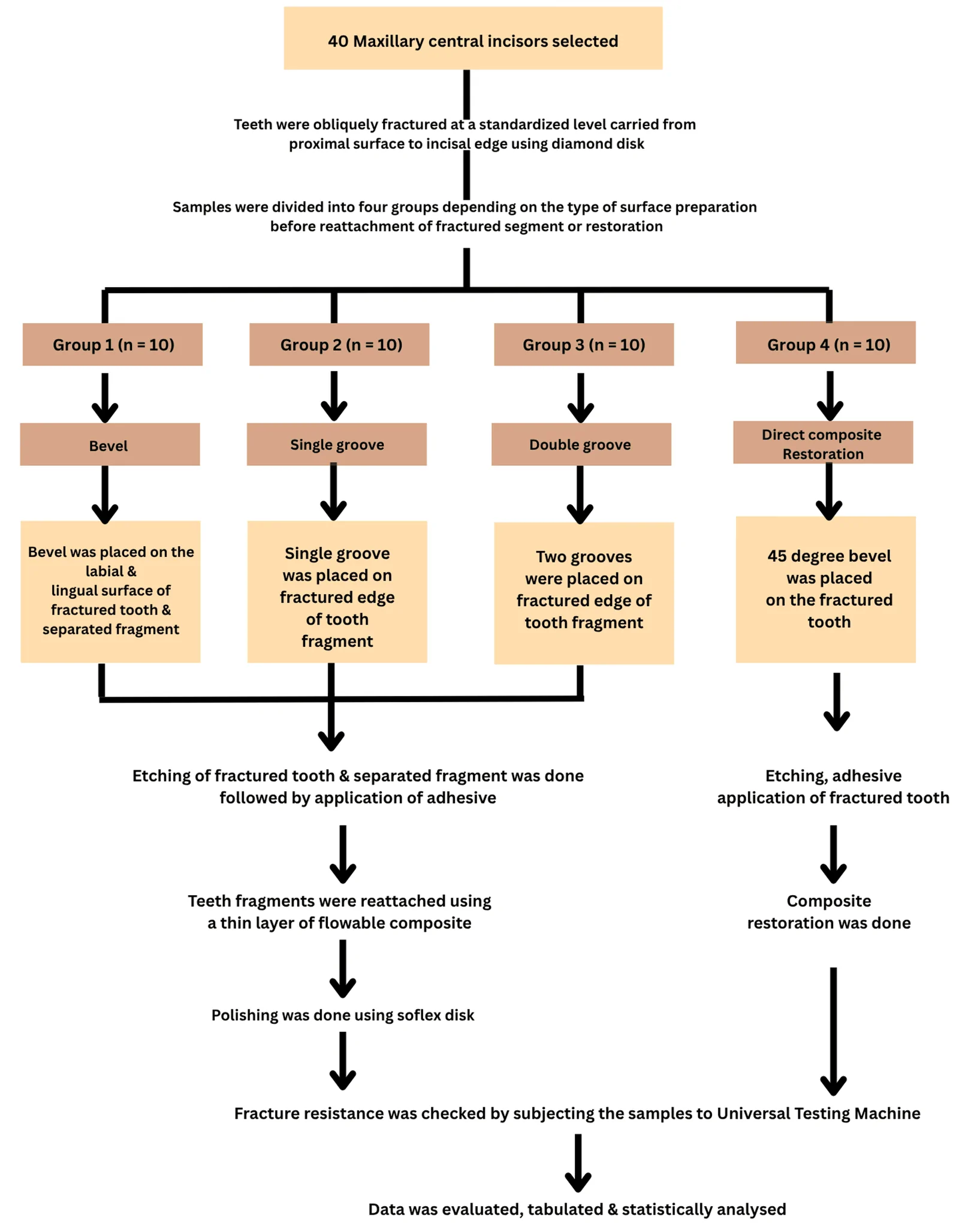
Study flowchart. Image created by the authors using Canva (Canva Pty Ltd., Surry Hills, NSW, Australia).

For all reattachment procedures, the fractured tooth surfaces and detached fragments were conditioned with 37% phosphoric acid etchant (Dentsply Sirona, Charlotte, NC) for 15 s, thoroughly rinsed with water for 20 s, and gently air-dried. A universal adhesive (Spectrum Bond Universal; Dentsply Sirona, Charlotte, NC) was then applied to both surfaces according to the manufacturer’s instructions, gently air-thinned, and polymerized using a light-curing unit (Endoking iCure; Endoking Dental Technology Co., Ltd., Shenzhen, China). A thin layer of microhybrid composite resin (Spectrum Universal Microhybrid Composite; Dentsply Sirona, Charlotte, NC) was placed between the fractured surfaces, after which the fragment was accurately repositioned under gentle finger pressure and light-cured from both the labial and palatal aspects. The irradiance of the light-curing unit was verified before specimen preparation using a calibrated dental radiometer and recorded as 1000 mW/cm². Irradiance was rechecked at regular intervals during specimen preparation to ensure consistency. For the direct composite restoration group, the missing incisal portion was reconstructed incrementally using the same composite resin following adhesive application, with each increment individually cured according to the manufacturer's recommendations. Upon completion of all restorations, finishing and polishing were performed using the Sof-Lex Finishing and Polishing System (3M ESPE, St. Paul, MN) to obtain standardized restoration contours and smooth surfaces (Figure 1). 

Standardization and fracture resistance testing

All specimen preparation, restorative procedures, finishing, polishing, and mechanical testing were performed by a single calibrated operator using identical instruments and standardized preparation dimensions to minimize the operator-dependent variability. The dimensions of the bevels and grooves were verified using a periodontal probe during specimen preparation. The universal testing machine (UTM) was calibrated according to the manufacturer's recommendations before testing. Following restoration, all specimens were stored in 0.9% normal saline for 24 hours. Each specimen was subsequently mounted in a UTM, and a compressive load was applied to the palatal aspect of the incisal edge at a crosshead speed of 1 mm/minute until a catastrophic fracture occurred. The maximum load at failure was automatically recorded in Newtons (N) and was considered the primary outcome measure.

Outcome measure

The primary outcome measure was the fracture resistance (N) of the restored teeth following mechanical loading. Fracture resistance values were compared among the bevel, single-groove, double-groove, and direct composite build-up techniques. The operator responsible for fracture testing was blinded to the restoration group allocation, and the specimens were coded before mechanical testing.

Statistical analysis

Statistical analyses were performed using statistical software (IBM SPSS Statistics for Windows, version 25.0; IBM Corp., Armonk, NY). Fracture resistance values were expressed as mean ± standard deviation (SD). The normality of the data distribution was assessed using the Shapiro-Wilk test. Intergroup comparisons were performed using ANOVA. When statistically significant differences were identified, Tukey's honestly significant difference (HSD) post hoc test was used for pairwise comparisons. A two-tailed *P* < 0.05 was considered statistically significant.

## Results

Forty extracted maxillary central incisors were evaluated, with 10 specimens allocated to each of the four experimental groups. Fracture resistance was compared among the bevel, single groove, double groove, and direct composite build-up techniques. One-way ANOVA demonstrated a statistically significant difference in fracture resistance among the four restoration techniques (*F* = 11.673, *P* < 0.001). The effect size (η² = 0.493) indicated that approximately 49.3% of the variance in fracture resistance was attributable to the preparation and restoration techniques used. The mean fracture resistance progressively increased from the bevel group (134.53 ± 28.85 N) to the single groove group (169.94 ± 43.52 N) and double groove group (198.60 ± 28.40 N), and was highest in the direct composite build-up group (239.23 ± 56.61 N), as shown in Table 1. The null hypothesis was therefore rejected.

**Table 1:** Intergroup comparison of fracture resistance (in Newtons) among the study groups. Data are presented as mean ± standard deviation (SD) and 95% confidence intervals (CIs). Intergroup comparisons were performed using one-way analysis of variance (ANOVA). Effect size is expressed as eta squared (η²) ^*^P < 0.05 was considered statistically significant.

Study groups	N	Mean ± SD	95% CI for mean		*F*-statistics	*P*-value	Effect size (η²)
			Lower bound	Upper bound			
Bevel	10	134.53 ± 28.85	113.894	155.175	11.673	<0.001*	0.493
Single groove	10	169.94 ± 43.52	138.806	201.073			
Double groove	10	198.60 ± 28.40	178.285	218.926			
Direct composite	10	239.23 ± 56.61	198.732	279.731			

The direct composite build-up group demonstrated significantly greater fracture resistance than the bevel group (mean difference = 104.70 N, *P* < 0.001) and the single-groove group (mean difference = 69.29 N, *P* = 0.002). Likewise, the double-groove technique showed significantly higher fracture resistance than the bevel technique (mean difference = 64.07 N, *P* = 0.005). However, no statistically significant differences were observed between the bevel and single-groove groups (*P* = 0.262), single-groove and double-groove groups (*P* = 0.523), or double-groove and composite build-up groups (*P* = 0.132), despite the numerical increase in mean fracture resistance values. Overall, the findings indicate that the direct composite build-up provided the highest fracture resistance, followed by the double-groove, single-groove, and bevel techniques. The incorporation of additional retentive features, particularly double-groove preparation, improved fracture resistance compared with bevel preparation alone, although direct composite restoration demonstrated the greatest reinforcement of fractured teeth (Table 2).

**Table 2:** Pairwise comparison of fracture resistance (in Newtons) among the study groups using Tukey's post hoc test. Pairwise comparisons were performed using Tukey's honestly significant difference (HSD) post hoc test following one-way analysis of variance (ANOVA). ^*^P < 0.05 was considered statistically significant.

Pairwise comparison	Mean difference (N)	95% CI of mean difference		*t*-statistics	*P*-value	Effect size (Cohen's *d*)
		Lower bound	Upper bound			
Bevel vs. single-groove	35.41	-14.04	84.86	1.929	0.262	0.863
Bevel vs. double-groove	64.07	14.62	113.52	3.491	0.005*	1.561
Bevel vs. direct composite	104.70	55.25	154.15	5.704	<0.001*	2.551
Single-groove vs. double-groove	28.66	-20.79	78.11	1.561	0.523	0.698
Single-groove vs. direct composite	69.29	19.84	118.74	3.774	0.002*	1.688
Double-groove vs. direct composite	40.63	-8.82	90.08	2.213	0.132	0.990

## Discussion

This in vitro study compared the fracture resistance of Ellis Class II fractured maxillary central incisors restored using bevel, single-groove, and double-groove preparations and direct composite build-up. A statistically significant difference was observed among the four techniques, with the direct composite build-up technique demonstrating the highest fracture resistance, followed by the double-groove, single-groove, and bevel techniques.

The superior performance of the direct composite build-up may be attributed to the increased bulk of composite resin and the absence of a fracture interface. Composite restorations distribute functional stresses more uniformly throughout the restored crown, thereby reducing stress concentration and improving resistance to fracture [11]. In contrast, fragment reattachment relies primarily on adhesive bonding across the fractured interface, which represents the weakest region during loading. These findings are consistent with those of Reis et al. [12] and Loguercio et al. [13], who also reported that composite build-up provided greater fracture resistance than conventional fragment reattachment techniques.

Among the reattachment techniques, double-groove preparation exhibited greater fracture resistance than single-groove and bevel preparations. The additional grooves increase the bonding surface area and provide mechanical interlocking, thereby enhancing stress distribution across the adhesive interface [14]. Although the double-groove group showed numerically higher retention values than the single-groove group, the difference was not statistically significant, suggesting that both groove designs improved retention compared with bevel preparation alone. Similar observations were reported by Loguercio et al. [13] and Sreen et al. [15], who demonstrated that internal groove preparations significantly improved the strength of reattached anterior teeth by enhancing mechanical retention.

Bevel preparation exhibited the lowest fracture resistance in the present study. Although beveling increases the enamel bonding area and improves marginal adaptation, it provides limited mechanical reinforcement because stresses remain concentrated at the adhesive interface. Comparable findings have been reported by Stellini et al. [16] and Demarco et al. [17], who concluded that bevel preparation alone cannot restore the original fracture resistance of intact teeth and that additional retentive preparations are required to improve mechanical performance.

The superior fracture resistance observed with direct composite build-up in the present study may be attributed to the increased bulk of the restorative material and more uniform stress distribution across the restored crown. However, the performance of fragment reattachment is influenced by the preparation design and adhesive material employed. In the present study, double-groove preparation improved fracture resistance compared with bevel preparation, suggesting that additional mechanical retention enhances the integrity of the bonded interface. In contrast, Pereira et al. [18] reported no significant difference in fracture resistance between fragment reattachment and direct composite restoration when contemporary adhesive materials, particularly preheated composite resins, were used. These findings indicate that the clinical success of fragment reattachment depends not only on the preparation design but also on the adhesive protocol and restorative material used.

Clinical implications

The findings of this study suggest that direct composite build-up provides the highest fracture resistance and may be preferred when the fractured fragment is unavailable. However, when the original fragment is available, fragment reattachment with double-groove preparation offers improved mechanical reinforcement while preserving natural tooth structure, esthetics, and function. These results may assist clinicians in selecting an appropriate restorative technique based on the clinical situation and availability of the fractured fragment.

Limitations

The present study was conducted under in vitro conditions and therefore could not fully replicate the complex oral environment, including thermal changes, cyclic masticatory loading, saliva, periodontal ligament support, and long-term aging of adhesive restorations. The relatively small sample size and assessment of immediate fracture resistance further limit the clinical extrapolation of the findings. In addition, fracture failure modes were not systematically classified as adhesive, cohesive, mixed, or restorable/non-restorable, which limits interpretation of the clinical nature of failure. Future studies incorporating thermocycling, mechanical fatigue, periodontal ligament simulation, detailed failure-mode analysis, different adhesive systems, and long-term clinical evaluation are recommended to validate and extend the present findings.

## Conclusions

The fracture resistance of restored Ellis Class II fractured maxillary central incisors differed significantly according to the preparation and restoration technique. Direct composite build-up demonstrated the highest fracture resistance, whereas double-groove preparation showed the greatest fracture resistance among the fragment reattachment techniques. The findings suggest that additional retentive features may enhance the mechanical performance of fragment reattachment under laboratory loading conditions. However, because this was an in vitro study evaluating immediate fracture resistance, the findings should not be interpreted as evidence of clinical superiority or long-term treatment success. Further studies incorporating thermomechanical aging, cyclic loading, and clinical follow-up are warranted to determine the clinical relevance of these findings.
